# Examining Erythrocytes as Potential Blood Biomarkers for Autism Spectrum Disorder: Their Relationship to Symptom Severity and Adaptive Behavior

**DOI:** 10.3390/biomedicines12112619

**Published:** 2024-11-15

**Authors:** Tomas Jasenovec, Dominika Radosinska, Ivan Belica, Barbara Raskova, Angelika Puzserova, Norbert Vrbjar, Jana Radosinska

**Affiliations:** 1 Institute of Physiology, Faculty of Medicine, Comenius University in Bratislava, Sasinkova 2, 811 08 Bratislava, Slovakia; ivan.belica@fmed.uniba.sk (I.B.); barbara.raskova@fmed.uniba.sk (B.R.); jana.radosinska@fmed.uniba.sk (J.R.); 2 Institute of Medical Biology, Genetics and Clinical Genetics, Faculty of Medicine, Comenius University in Bratislava, Sasinkova 4, 811 08 Bratislava, Slovakia; dominika.radosinska@fmed.uniba.sk; 3 Centre of Experimental Medicine, Slovak Academy of Sciences, Dúbravská Cesta 9, 841 04 Bratislava, Slovakia; angelika.puzserova@savba.sk (A.P.); norbert.vrbjar@savba.sk (N.V.)

**Keywords:** autism spectrum disorder, ADOS-2, VABS-3, erythrocytes, erythrocyte deformability, nitric oxide, osmotic resistance, Na,K-ATPase

## Abstract

Background: Multiple research teams have documented various abnormalities in erythrocyte properties in children with autism spectrum disorder (ASD) compared with neurotypical individuals. Reduced erythrocyte deformability, a crucial factor for microcirculation and oxygen delivery, may affect brain function. Other key factors like nitric oxide (NO) and Na,K-ATPase-regulated cation transport also play roles in both erythrocyte deformability and ASD, suggesting a possible relationship between erythrocyte parameters and autism severity. Thus, this study aims to describe these associations, exploring erythrocyte properties as potential biomarkers in ASD. Methods: A total of 179 ASD children were enrolled in this study. Diagnosis was confirmed by the Autism Diagnostic Observation Schedule—Second Edition (ADOS-2) and Autism Diagnostic Interview-Revised. The Vineland Adaptive Behavior Scales, Third Edition (VABS-3), was used to assess adaptive behavior. RBC deformability was measured using a filtration technique, while NO production by RBCs was assessed via DAF-2DA fluorescence. Na,K-ATPase kinetics and RBC osmotic resistance were evaluated spectrophotometrically. Results: Children with more severe ASD symptoms had more impaired deformability and osmotic resistance than children with mild symptoms. Higher RBC NO production was linked to better scores in some VABS-3 subdomains, and in the social affect domain of ADOS-2. Higher affinity of Na,K-ATPase for sodium negatively correlated with the occurrence of repetitive and restricted behavior—one of the core ASD symptoms. Conclusions: This study identified potential links between ASD severity and RBC properties. While erythrocyte quality can influence ASD symptomatology, the observed relationships—such as those involving RBC deformability, NO production, Na,K-ATPase kinetics, and osmotic resistance—were not strong or consistent enough to be considered reliable diagnostic or prognostic biomarkers.

## 1. Introduction

Autism Spectrum Disorder (ASD) refers to a group of conditions that typically manifest before the age of three years, characterized by challenges in social interaction, communication, and restricted, stereotypical, and repetitive behaviors [1]. In addition to these core symptoms, children with ASD often experience various physical comorbidities, such as immunological, gastrointestinal, or neurological issues [2]. Although the etiology of ASD is intensively studied, it remains uncertain. While ASD has a high degree of heritability [3], numerous environmental factors, as well as gene–environment interactions, may contribute to its development, including parental age, preterm birth, breastfeeding duration, exposure to toxic metals, and endocrine disruptors [4,5,6,7,8].

The prevalence of ASD is approximately 1% of the population, with boys being four times more likely to develop the condition [9]. The higher prevalence of ASD in males is linked to prenatal testosterone effects (“extreme male brain” theory) [10] and the “female protective effect”, which suggests females need a higher genetic load in order for ASD to develop [11]. Diagnosis of ASD relies on assessing specific behavioral traits in children with ASD, and requires collaboration between pediatricians, psychologists, and psychiatrists. Given that this process is both time- and resource-demanding, in recent years, several potential biomarkers have been identified that could be used to detect children at risk before disease onset, support early diagnosis for timely intervention, categorize patients by symptom severity, and predict therapeutic efficacy. Among the recognized mechanisms involved in ASD etiopathogenesis, commonly accepted biomarkers include those indicating inflammatory response [12], mitochondrial dysfunction [13], oxidative stress [12,14,15], altered inhibitory/excitatory signaling due to gamma-aminobutyric acid and glutamate imbalances [16], and cerebral hypoperfusion [17]. Research has shown that the presence of damaged red blood cells (RBCs) can impair cerebral blood flow and prolong capillary transit time [18]. Oxidatively damaged RBCs have also been associated with microglial activation and cerebral microhemorrhages [19], highlighting the critical role of RBC functional status in maintaining proper microcirculation.

The properties of RBCs in individuals with ASD have attracted many research groups, as several RBC parameters have been found to be altered in comparison with neurotypical individuals. Abnormalities in erythrocyte shape, which are a typical feature in ASD [20], were also found to be mostly reversible, as their morphology was restored after 24 h of incubation with antioxidants [21]. Additionally, children with ASD exhibit reduced RBC deformability compared with neurotypical children [22,23]. This characteristic represents the ability of the RBCs to change their shape without sustaining damage, and has a significant impact on both hemodynamics and RBC lifespan [24]. RBCs also participate in blood flow regulation through several mechanisms, including the release of ATP [25] and the production of nitric oxide (NO). RBCs possess their own endothelial NO synthase, enabling them not only to absorb excess NO from circulation but also to contribute significantly to NO bioavailability in the blood [26]. NO plays a key role in maintaining RBC deformability [27]. Studies have noted higher plasma and RBC NO levels in individuals with ASD compared to their neurotypical counterparts [23,28,29,30]. One of the key factors influencing the surface-to-volume ratio of erythrocytes, and thus RBC deformability, is their ability to maintain ionic and fluid balance, a process in which Na,K-ATPase plays a central role. Na,K-ATPase is also involved in regulating another important aspect of RBC deformability—cytoplasmic viscosity [31]. The activity of Na,K-ATPase in ASD also seems to be affected, though available studies contradict each other [23,32]. Mean cell volume (MCV) is one of the most commonly measured blood parameters and is routinely used to investigate the causes of anemia. Studies show that MCV tends to increase with age, while the size of aging RBCs gradually decreases. Interestingly, RBCs’ sizes often correlated with their deformability [33,34].

In summary, the functional properties of RBCs are essential for maintaining adequate microcirculation in all tissues, including the brain, and numerous studies have identified their deterioration in individuals with ASD. This study is built on previous research [23] that confirmed the earlier findings mentioned above and identified new RBC characteristics that differ between neurotypical and ASD children, such as RBC deformability, NO production, RBC osmotic resistance, and Na,K-ATPase activity in RBC membranes. This raises the question of whether the deterioration of RBC functional properties, potentially affecting microcirculation, contributes to ASD symptomatology, or if these changes merely reflect broader physiological alterations associated with ASD. Thus, the aim of this research is to investigate whether there is a relationship between erythrocyte properties and the severity of clinical symptoms in children with ASD. Our findings could help to verify the potential role of RBC parameters as biomarkers or prognostic indicators for this condition.

## 2. Materials and Methods

### 2.1. Study Design

In this study, we enrolled 179 non-verbal children diagnosed with ASD (147 boys and 32 girls). The median age of the children with ASD was 3.5 years, ranging from 2.6 to 8.1 years. ASD was diagnosed based on the DSM-V criteria [1]. The diagnostic assessments used in this study included the Autism Diagnostic Observation Schedule—Second Edition (ADOS-2) and the Autism Diagnostic Interview—Revised, as outlined in prior research [23,35]. All children suspected of having ASD underwent Module 1 of the ADOS-2, which is intended for children over 30 months of age who do not regularly use phrase speech. Module 1 consists of 10 structured activities that evaluate social interaction and communication deficits, with specific behavioral criteria required for an ASD diagnosis. Children were required to have shown no signs of infectious disease for at least two weeks prior to undergoing any diagnostic procedures.

Detailed information regarding the use of nutritional supplements and medications by mothers during pregnancy, by fathers at the time of conception, as well as those taken by children, is provided in Supplementary Table S1. This table also includes additional data, such as parents’ ages at childbirth, the child’s birth weight and length, lactation duration, and details on smoking habits before pregnancy, at conception, and throughout the pregnancy. To summarize, the average age of mothers at childbirth was 32 years, and 34 years for fathers. Approximately 3% of mothers used antidepressants during pregnancy, while 18% reported an atopic disorder (1.5% with asthma). Additionally, 37% of mothers reported experiencing an infectious disease during pregnancy. Among fathers, 10% reported an atopic disorder (4% with asthma). Regarding the children, 51% were delivered by cesarean section, and 18.5% had an atopic disorder (1.5% with asthma). Before pregnancy, 25% of mothers smoked cigarettes, decreasing to 4% during pregnancy and increasing to 18% by the time of examination. At the time of conception, 26% of fathers were smoking.

All assessments and diagnostic procedures were carried out at the Institute of Physiology, Faculty of Medicine, Comenius University, Bratislava, at the Academic Research Center for Autism. The study received approval from local authorities, specifically the Ethics Committee of the Faculty of Medicine, Comenius University, and the University Hospital in Bratislava, Slovakia. The research adhered to the principles of the Declaration of Helsinki. Informed consent was obtained from and signed by all caregivers of all children enrolled in the study. All procedures were carried out in line with previous study [23].

### 2.2. ASD Symptom Assessment

To assess autistic symptoms, we used the ADOS-2, a semi-structured, standardized assessment designed to evaluate social interaction, communication, and play behaviors in individuals suspected of having ASD or other pervasive developmental disorders. The ADOS-2 diagnostic algorithm focuses on two core behavioral domains: social affect (SA) and restricted and repetitive behavior. The SA domain assesses difficulties in communication and reciprocal social interaction, which may include reduced use of gestures, difficulty pointing at distant objects, unusual eye contact, challenges in responding to joint attention, problems with showing objects or toys to another person, or variability in the quality of social responses to the examiner or parent. The “restricted and repetitive behavior” domain evaluates behaviors such as the stereotypical or idiosyncratic use of words, sensory examination of play material or another person, unusual repetitive movements or posturing, or insistence on unusual routines. These behaviors are systematically scored, contributing to the overall assessment of ASD-related symptoms. Calibrated severity scores (CSS) on a scale of 1 to 10 were calculated for each participant. Scores between 8 and 10 indicate a high level of autistic symptoms, scores of 5 to 7 suggest mild symptoms, scores of 3 to 4 represent low symptom levels, and scores of 1 to 2 indicate minimal or no symptoms [36].

### 2.3. Assessment of Adaptive Behavior

To assess adaptive functioning, the Vineland Adaptive Behavior Scales, Third Edition (VABS-3), were utilized [37]. The VABS-3 are administered via a semi-structured interview conducted with the children’s primary caregivers, focusing on adaptive behaviors. These behaviors refer to the ability to perform tasks essential for daily life, or an individual’s capacity to meet social and independent living standards [37]. The VABS-3-tool measures three main domains, each comprising three subdomains: communication (receptive, expressive, and written communication), daily living skills (personal, domestic, and community skills), and socialization (interpersonal relationships, play and leisure, and coping skills). Raw scores were converted into standard scores, facilitating comparison across various age groups. Typically, VABS-3 scores in each subdomain have a mean of 15 and a standard deviation of 3. Scores between 13 and 17 are considered to be within the normal range, while scores below 12 indicate a lower range of ability. Higher scores reflect better adaptive functioning. Receptive communication involves listening and understanding responses to gestures, words, requests, and instructions, as well as comprehending their meaning. Expressive communication encompasses the use of sounds, words, sentences, and gestures. Writing involves working with the alphabet, words, and text. Personal skills include self-care activities such as feeding, maintaining hygiene, and clothing. Domestic skills encompass household tasks, including adhering to safety precautions, maintaining order, and ensuring hygiene. Community skills involve abiding by social norms, effectively managing finances, and utilizing various means of communication. Play and leisure activities promote engagement, imitation, and group dynamics. Relationships with others, both within family settings and beyond, involve gestures, emotional expressions, and conversations. Coping skills requires adaptability and politeness in social interactions.

### 2.4. Blood Processing

After the diagnostic procedures, venous blood was collected into EDTA-containing vacutainer tubes and was centrifuged at 1150× *g* for 5 min at 4 °C. The buffy coat and upper 20% of RBCs were discarded. The remaining RBCs were washed three times with saline solution and were immediately processed and used for measurements if not stated otherwise.

### 2.5. Erythrocyte Deformability Measurements

RBC deformability was evaluated using a filtration technique [35], which measured the ratio of RBCs passing through a 5 µm pore filter (Ultrafree-MC SV Centrifugal Filter; Merck Millipore Ltd., Tullagreen Carrigtwohill, Ireland) compared to the initial RBC count. RBC count and MCV were determined using a Sysmex hematological analyzer (Sysmex F-820, Sysmex Corp, Tokyo, Japan) [38].

### 2.6. Erythrocyte Nitric Oxide Production

RBC NO production was assessed using specific fluorescent probe (DAF-2 DA, 25 µM, ab145283, Abcam, Cambridge, UK). The whole blood was diluted in a modified saline (in mM: NaCl 119, KCl 4.7, NaHCO_3_ 25, MgSO_4_·7H_2_O 1.17, KH_2_PO_4_ 1.18, CaCl_2_·2H_2_O 2.5, Na_2_EDTA 0.03, glucose 5.5, and pH 7.4) and incubated with fluorescent probe at room temperature in the dark for 10 min. Fluorescence signals were captured using an Axiolab 5 microscope (Zeiss, Jena, Germany) and analyzed using ImageJ 1.53e software (National Institutes of Health, Bethesda, MD, USA) as previously [38].

### 2.7. Assessment of Erythrocyte Osmotic Resistance

Osmotic resistance was evaluated by exposing washed RBCs to NaCl solutions ranging from 0% to 0.9% for 30 min. After centrifugation, the extent of hemolysis in supernatants was spectrophotometrically measured at λ = 540 nm. Supernatants from the 0.9% NaCl solution were considered nonhemolytic, serving as a baseline for no hemolysis, while supernatants from distilled water were used as the reference standard for 100% hemolysis. IC_50_ values, indicating the NaCl concentration at which 50% hemolysis occurred, were calculated from the obtained data [39].

### 2.8. Erythrocyte Membrane Isolation and Na,K-ATPase Enzyme Kinetics Measurements

Washed RBCs were homogenized in 50 mM TRIS buffer (7.4 pH) and centrifuged at 13,000× *g* for 30 min at 4 °C. Afterwards, the supernatant was discarded and membrane-containing pellets were repeatedly homogenized and centrifuged in 30, 20, and 10 mM TRIS buffer to remove residual hemoglobin. The protein content in each sample was determined by Lowry’s method [40]. Na,K-ATPase activities were measured within 2–100 mM Na^+^ and constant presence of K^+^, Mg^2+^ and ATP (10, 4, and 8 mM, respectively). An aliquot without Na^+^ and K^+^ was used to differentiate activities of the other Mg-dependent ATPases. RBC membrane proteins (50 µg) were preincubated for 20 min at 37 °C, and ATP was added. After 20 min, the reaction was halted by the addition of 12% trichloroacetic acid. Inorganic phosphate generated via ATP hydrolysis was spectrophotometrically measured at λ = 700 nm [41]. From obtained data, the kinetic curves were drawn and the following kinetic parameters were calculated: V_max_ (maximum velocity of the reaction—represents a number of active Na,K-ATPase molecules) and K_Na_ (Na^+^ concentration required for ½ V_max_—represents Na^+^ binding ability of the enzyme). 

### 2.9. Statistical Analyses

Data normality was assessed using the D’Agostino–Pearson test. Data are presented as means ± standard deviations or medians with interquartile ranges for non-Gaussian distributions and ordinal data. Outliers were identified using the Grubbs test and excluded from further analyses. Spearman’s correlation coefficient was used to detect potential relationships between RBC parameters and the individual score of ADOS-2 and VABS-3 tools. To account for multiple comparisons, the Benjamani–Hochberg procedure was applied with the false discovery rate (FDR) threshold set to 0.2. Potential sex differences were analyzed using an unpaired *t*-test or a Mann–Whitney test when appropriate. Similar comparison was made for RBC parameters between children with a high level of autistic symptoms (CCS 8–10) and children with mild symptoms (CSS 5–7). Statistical significance was set at *p* < 0.05. Data analysis was performed using Microsoft Excel, SigmaPlot 13 (Grafiti LLC, Palo Alto, CA, USA), and GraphPad Prism 7.02 software (GraphPad Software, San Diego, CA, USA).

## 3. Results

This study focused on examining the relationships between several RBC parameters—deformability, osmotic resistance, MCV, nitric oxide production, and Na,K-ATPase kinetic parameters—with the following ADOS-2 items: social affect, restrictive and repetitive behavior, and calibrated severity score. Additionally, RBC parameters were correlated with nine subdomains of VABS-3: (receptive, expressive, written, personal, domestic, community, interpersonal relationships, play and leisure time, and coping skills). An overview of the measured parameters is presented in Table 1. In addition to correlational analyses, we used a *t*-test to compare the RBC parameters between children with a high level of autistic symptoms (CSS 8–10) and those with a mild level of autistic symptoms (CSS 5–7).

**Figure 1:**
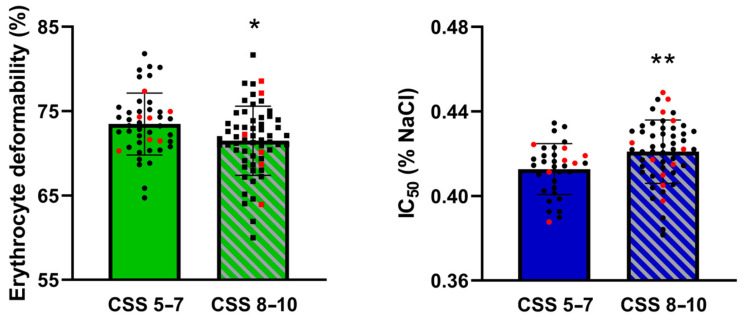
Erythrocyte deformability and osmotic resistance in children with a mild (CSS 5–7) and high (CSS 8–10) level of autism symptoms. Abbreviations: CSS—calibrated severity score, IC_50_–NaCl concentration at which 50% hemolysis occurred, * *p* < 0.05, ** *p* < 0.01 vs. CSS 5–7. Black dots signify male study participants, while red dots represent females.

A total of 78 statistical tests were conducted, including 72 correlations of RBC parameters with ADOS-2 and VABS-3 and 6 *t*-tests comparing RBC parameters between children with high and mild levels of autism symptoms. Of these tests, 15 (13 correlations and 2 *t*-tests) were initially considered statistically significant before the Benjamani–Hochberg procedure was applied. The *t*-tests revealed that children with a high level of autism symptoms had significantly lower RBC deformability (*p* = 0.012) and impaired osmotic resistance (*p* = 0.008) compared to those with mild symptoms (Figure 1), and this remained significant after Benjamani–Hochberg correction. A list of the significant correlations is provided in Table 2. After the application of the Benjamani–Hochberg correction, 6 of the 13 correlations remained statistically significant.

Regarding RBC NO production, only two correlations remained statistically significant after correction (one being personal and the other being play and leisure time). However, even among the correlations that were not retained, a pattern appears to emerge: in all cases, children with higher RBC NO production tended to score closer to normal values on the VABS-3 and had a lower score on the social affect domain of ADOS-2, indicating less severe ASD symptoms. Although the correlations between RBC NO production and ASD symptomatology may be considered borderline significant (even before the Benjamani–Hochberg correction), the likelihood that all of these correlations would randomly point in the same direction seems low.

In addition to RBC NO production, children with worsened osmotic resistance were found to have higher levels of autism symptoms, as indicated by the CSS score. Furthermore, better RBC deformability was associated with better scores in the community (“daily living skills” subdomain) and written communication. However, the significance of the latter association was lost after correcting for multiple comparisons.

Children with lower MCV exhibited higher calibrated severity scores and more severe restricted and repetitive behaviors, as measured by ADOS-2, though these correlations were weak and did not remain significant after correction. Interestingly, the latter was similarly negatively correlated with K_Na_, which represents the Na^+^-binding properties of Na,K-ATPase.

There were no differences in RBC parameters, ADOS-2, and VABS-3 subscales between girls and boys, and no effect of age on RBC parameters (other than MCV, which is known to increase with age) was detected (data available in Supplementary Table S2).

## 4. Discussion

RBCs play a critical role in oxygen transport, and any deterioration in their function can impair tissues and organs, particularly those with an oxygen demand as high as that of the human brain. Since RBC deformability is crucial for proper microcirculation, impaired RBC deformability may have a negative impact on the developing brain, potentially influencing the behavior of children with ASD. Previous research has shown that RBC deformability is significantly lower in children with ASD [22,23]. However, aside from a preliminary study [35], no other studies have specifically examined the relationship between RBC deformability and the severity of ASD symptoms. In the present study, children with ASD who had lower RBC deformability showed impairments in certain VABS-3 categories (written communication and community part of daily living skill). RBC deformability was also more impaired in children with a high level of autism symptoms when compared to the children with mild symptoms.

In RBCs of autistic patients, higher Na,K-ATPase activity has been observed, although its sodium-binding properties remain unchanged [23]. This aligns with findings in the frontal cortex and cerebellum of autistic children, where increased Na,K-ATPase activity has been suggested as a response to elevated cellular calcium levels [42]. A possible mechanism influencing Na,K-ATPase activity may be related to the energy metabolism of RBCs. Since RBCs lack mitochondria, they rely on anaerobic glycolysis for ATP production. In ASD patients, a lower plasma lactate/pyruvate ratio has been observed [43], which promotes a higher glycolytic influx in RBCs. An increase in both absolute and relative pyruvate levels enhances ATP synthesis [44,45], ensuring abundant substrate availability to support Na,K-ATPase activity. Additionally, the mechanosensitive Piezo1 channel may also play a role. More frequent activation of Piezo1, which could be assumed in the condition of decreased RBC deformability in ASD children [22,23], increases cell membrane permeability to calcium ions, which then activates Gardos channels, regulating potassium ion permeability and resulting in RBC shrinkage [46]. Conversely, Piezo1 inhibition leads to cell swelling and reduced osmotic resistance under hypotonic conditions [47,48]. Interestingly, Piezo1 activation also enhances Na,K-ATPase-mediated ion transport in mouse lens, though this has not yet been documented in RBCs [49].

Similarly to Piezo1 channel, another potential mechanism involves altered glutamate metabolism in ASD individuals. Elevated glutamate levels in the blood of individuals with autism [50], could activate corresponding receptors (*N*-methyl-d-aspartate—NMDA-receptors) present in RBC membranes, increasing intracellular calcium and leading to cell shrinkage, as well as an increase in osmotic resistance [51]. Gunes et al. [52] reported that children with autism have lower MCV than neurotypical children, although there is no consensus on this finding, as other studies reported no changes in MCV in ASD [20,23]. In this study, a lower erythrocyte volume in autistic children appears to be associated with more severe repetitive and restrictive behavior, as well as a calibrated severity score according to the ADOS-2 procedure. This could be related to altered Na,K-ATPase function, as the sodium-binding properties of this enzyme (indicated by the K_Na_ value) were enhanced in children with more severe repetitive and restrictive behaviors. The observed increase in Na,K-ATPase activity in ASD conditions [23] may serve as a compensatory response to counteract potential ion imbalances.

Regarding the improved osmotic resistance observed in autism [23], there are several possible mechanisms which may be responsible. Firstly, the increase in osmotic resistance in ASD might be caused by more rigid RBC membranes as a result of increased oxidative stress, which is present in ASD [23]. While oxidative stress generally induces hemolysis, it has also been observed to increase osmotic resistance after exposure to strong oxidants as a consequence of increased RBC membrane stiffness [53]. Another possible mechanism includes the activation of Piezo1 or NMDA receptors, leading to RBC dehydration and increasing RBCs’ capacity to resist hypotonic conditions [47,48,51]. It is worth noting that in cases of cell shrinkage, the improvement in osmotic resistance is likely primarily related to hypotonic conditions, in which it is typically measured, rather than to hypertonic environments. In a previous study [23], the osmotic resistance in ASD was generally increased; however, its IC_50_ values were more scattered, with some individuals having worsened osmotic resistance compared with neurotypical children. In this study, greater ASD severity, including increased repetitive and restricted behaviors and higher overall ADOS-2 scores, was associated with worsened RBC osmotic resistance. This variability could be explained by the complex, possibly dose-dependent effects of oxidative stress on the RBC membrane.

Nitric oxide appears to play a pivotal role in neurodevelopmental diseases such as ASD. As mentioned, both plasma and RBC levels of NO were shown to be elevated in individuals with ASD [23,28,29,30], which confirms the involvement of this gasotransmitter in this disorder’s pathophysiology. Excessive NO, through the formation of reactive nitrogen species, and in combination with reactive oxygen species, can lead to mitochondrial dysfunction, oxidative stress, and neuroinflammation [54]. A link has been found between NO plasma levels and interferon-γ activity in ASD, which induces the expression of inducible NO synthase [55,56]. Additionally, the severity of autism has been positively correlated with the nitration of low-sulfur protein residues in the hair of children with ASD [57]. In another study, increased nitrate levels in saliva were observed in children with ASD, though no significant relationships were found between serum or saliva nitrate levels and ASD severity [58]. Research utilizing mouse models of ASD has also revealed interesting findings—in Shank3 and Cntnap2 models, inhibition of neuronal NO synthase (nNOS) reduced nitrosative stress and alleviated ASD-like symptoms, positioning nNOS as a potential therapeutic target [59]. Conversely, in the valproic acid-induced mouse model of ASD, nNOS expression was decreased in the basolateral amygdala, while its restoration led to a reduction in ASD-like behaviors [60]. Interestingly, activation of both Piezo1 and NMDA receptors has been shown to facilitate NO production in RBC [51,61]. The results of this study suggest that, contrary to the generally accepted negative aspect of NO signaling in ASD, increased production of NO in RBCs appears to be beneficial. Children with higher RBC NO production exhibited several VABS-3 behavioral scores closer to age-normative values. It is also important to mention that an excessively high concentration of NO (e.g., from activated polymorphonuclear leukocytes) may result in decrease in erythrocyte deformability [62]. It is also worth noting that the RBC produces NO by endothelial NO synthase, not by nNOS. The relationship between NO levels and ASD symptom severity may vary depending on the specific tissue or organ involved, and could be influenced by the dominant underlying etiology of ASD in particular individuals. Therefore, further research is necessary to clarify these dynamics.

It is also appropriate to mention that certain parameters, such as MCV, might be affected by other factors that are commonly observed in ASD children, e.g., various hypovitaminoses, iron deficiencies (especially in children that tolerate only certain types of food) [63,64], or heavy metals exposure [65]. These variables may not be present uniformly across all autistic populations, and thus may not appear uniformly in studies involving children with ASD, potentially contributing to the inconsistency in findings presented by different research teams.

Despite ASD being more prevalent in boys than girls, this study does not indicate significant differences in RBC parameters or ADOS-2 and VABS-3 scores between sexes. This aligns with previous research [23] and a recent meta-analysis, which found minimal, non-significant sex differences across domains, except for a potential higher prevalence of repetitive and restricted behaviors in males [66].

## 5. Conclusions

In our study, we identified potential relationships between the severity of autism and the functional parameters of erythrocytes. While it is likely that erythrocyte properties play a role in the etiopathology of ASD, the relationships we observed—such as those involving erythrocyte deformability, NO production, Na,K-ATPase function, and osmotic resistance—were too weak and inconsistent to be definitively considered as diagnostic biomarkers or reliable predictors of this disease. Further research is needed to clarify the potential role of erythrocytes in ASD and to determine if these functional parameters could have clinical relevance in the future.

## Figures and Tables

**Table 1:** Erythrocyte parameters, scores in ADOS-2 and VABS-3 in children with ASD.

	Mean ± SD
Erythrocyte Parameter	
Deformability (%)	72.3 ± 4.1
Osmotic resistance-IC_50_ (% NaCl)	0.418 ± 0.014
Mean cell volume (fl)	77.1 ± 4.5
Nitric oxide production (a.u.)	986 ± 106
V_max_ (µM Pi/mg protein/h)	2.66 ± 0.71
K_Na_ (mM NaCl)	30.5 ± 13.7
	median (IQR)
**ADOS-2 parameter**	
Social affect	7 (6–8)
Repetitive and restricted behavior	9 (7–10)
Calibrated severity score	8 (7–9)
	
**VABS-3 parameter**	
**Communication**	
-Receptive	5.5 (3–8)
-Expressive	5 (3–8)
-Written	11 (8–12)
**Daily living skills**	
-Personal	9 (8–11)
-Domestic	11 (9–12)
-Community	9 (8–10)
**Socialization**	
-Interpersonal relationships	8 (7–9)
-Play and leisure time	8 (6–10)
-Coping skills	8.5 (8–10)

Abbreviations: ADOS-2—Autism Diagnostic Observation Schedule—Second Edition; ASD—autism spectrum disorder; a.u.—arbitrary units; IC_50_—NaCl concentration at which 50% hemolysis occurred; IQR—interquartile range; K_Na_—NaCl concentration required for ½ maximal activation of Na,K-ATPase; SD—standard deviation; VABS-3—Vineland adaptive behavior scales, third edition; and V_max_—maximal velocity of reaction.

**Table 2:** Significant correlations between erythrocyte parameters and scores obtained in ADOS-2 and VABS-3.

	ADOS-2/VABS-3 Tool	Erythrocyte Parameter	*p* Value (Unadjusted)	Spearman’s R	N	Significance (FDR = 0.2)
VABS-3	Written	Deformability	0.029	0.34	41	no
	Community	Deformability	0.007	0.41	41	yes
	Expressive	Nitric oxide	0.037	0.35	36	no
	Personal	Nitric oxide	0.020	0.39	36	yes
	Domestic	Nitric oxide	0.043	0.40	26	no
	Interpersonal relationships	Nitric oxide	0.038	0.35	36	no
	Play and leisure time	Nitric oxide	0.014	0.41	36	yes
ADOS-2	Social affect	Nitric oxide	0.038	−0.25	70	no
	Calibrated severity score	Osmotic resistance	0.006	0.29	89	yes
	Repetitive and restricted behavior	Osmotic resistance	0.009	0.27	89	yes
	Repetitive and restricted behavior	Mean cell volume	0.047	−0.19	106	no
	Calibrated severity score	Mean cell volume	0.034	−0.21	106	no
	Repetitive and restricted behavior	K_Na_	0.018	−0.29	67	yes

Abbreviations: ADOS-2—Autism Diagnostic Observation Schedule—Second Edition; FDR—false discovery rate; K_Na_—NaCl concentration required for ½ maximal activation of Na,K-ATPase, VABS-3—Vineland adaptive behavior scales, third edition. Osmotic resistance is represented by the IC_50_ value, defined as the NaCl concentration at which 50% hemolysis occurs. Therefore, a higher IC_50_ value indicates lower osmotic resistance.

## Data Availability

The data supporting the findings of this study are available in this article.

## Supplementary Materials

The following supporting information can be downloaded at https://www.mdpi.com/article/10.3390/biomedicines12112619/s1. Table S1—Characteristics of study participants and their parents and Table S2—Sex differences in erythrocyte, ADOS-2 and VABS-3 parameters in children with autism spectrum.
